# Feasibility and Acceptability of Collaborative Augmented Reality for Older Adults and Companions: Protocol for a Randomized Controlled Trial

**DOI:** 10.2196/83864

**Published:** 2026-02-11

**Authors:** Nilanjan Sarkar, Judith A Tate, Mahrukh Tauseef, Akshith Ullal, Cathy A Maxwell, Lisa A Juckett, Douglas W Scharre, Alai Tan, Rendong Zhang, Zalen Ingram, Lorraine C Mion

**Affiliations:** 1 Mechanical Engineering and Computer Science School of Engineering Vanderbilt University Nashville, TN United States; 2 College of Nursing The Ohio State University Columbus, OH United States; 3 Electrical and Computer Engineering School of Engineering Vanderbilt University Nashville, TN United States; 4 Intelligent Clinical Care Center University of Florida Gainesville, FL United States; 5 College of Nursing University of Utah Salt Lake City, UT United States; 6 School of Health & Rehabilitation Sciences College of Medicine The Ohio State University Columbus, OH United States; 7 Division of Cognitive Neurology, Department of Neurology College of Medicine The Ohio State University Columbus, OH United States

**Keywords:** older adults, loneliness, photorealistic avatars, interactive communication technology, social connection

## Abstract

**Background:**

Loneliness and social isolation are common among older adults and are associated with dire consequences. Studies using interactive communication technology (ICT) interventions with older adults have yielded mixed results. Advancements in collaborative head-mounted display augmented reality (HMD-AR) can provide older adults who are geographically distant from their families with a more diverse range of interactive activities, thus offering greater potential to enhance social connection.

**Objective:**

We examined the feasibility and acceptability of cocreated collaborative HMD-AR activities and 2D ICT (eg, Zoom) activities among older adults and their family members and close friends.

**Methods:**

In total, 8 pairs of older adults and their designated companion (family or friend) from the greater Nashville area were randomized to the HMD-AR or 2D ICT groups. Eligibility criteria for older adults included being 60 years or older, being able to tolerate HMD-AR, being cognitively and physically able to participate, and having a companion willing to participate. For long-term care settings, participants must have been a resident for at least 3 months. All participants lived within a 1-hour driving distance from the investigators’ university. Each older adult–companion pair participated in eight 30-minute sessions over 4 weeks. Participants randomized to the HMD-AR group had photorealistic avatars created; they participated in collaborative activities (ie, fireplace decoration, checkers) that were cocreated in an earlier study. Those randomized to the 2D ICT group had the opportunity to play virtual checkers or house décor games. Engineers remained on-site for all participants to assist as needed. The primary outcome was the feasibility of the study processes (ie, recruitment, retention, and data collection) and the technology (ie, viability, usability, comfort, ease of use, and acceptability). The study used face-to-face questionnaires and observations to collect data.

**Results:**

Funding was received from the National Institute on Aging in August 2022 and from the National Science Foundation in October 2022. This study was approved by the local institutional review board. Following the initial design and testing of the HMD-AR activities, recruitment and data collection for the feasibility and acceptability study began in April 2024 and were completed in May 2025. Of the 8 enrolled older-companion pairs, 8 (100%) completed all sessions. The final data acquisition has been completed, and data cleaning is currently ongoing. Results are intended to be published in 2026.

**Conclusions:**

To our knowledge, this study is the first collaborative augmented reality study using photorealistic avatars between older adults and their family members or friends. Our study will determine whether the use of HMD-AR is feasible, and the results of this pilot study will inform a full-scale randomized controlled trial evaluating the efficacy of the intervention to reduce loneliness among older adults.

**Trial Registration:**

ClinicalTrials.gov NCT06179225; https://clinicaltrials.gov/study/NCT06179225

**International Registered Report Identifier (IRRID):**

DERR1-10.2196/83864

## Introduction

### Background

#### Loneliness and Social Isolation Are Common Health Problems Among Older Adults

Social connection is a critical health determinant in preventing loneliness (the subjective feeling of being alone or isolated) and social isolation (the objective measure of social contact, communication, or social activities), both of which are common health problems among older adults [1-4]. Among community-dwelling older adults, up to one-fourth experience social isolation, and up to 46% report feeling lonely [2-4]. Among older adults residing in long-term care (LTC) facilities, such as assisted living, social isolation occurs in 4% of cases, moderate loneliness in 61%, and severe loneliness in 35% [5-8]. Social isolation and loneliness have significant adverse consequences, including increased mortality, cardiovascular disease, depression, suicide, cognitive and physical decline, reduced quality of life, and increased health care use [4,9-11]. Numerous recommendations have been made to alleviate social isolation and loneliness among older adults, which include technology-based solutions [3,4,12].

#### Social Presence as the Underlying Mechanism in Effective Interactive Communication Technology

Interactive communication technology (ICT) interventions, such as social media and video-mediated visits, have been examined for their effects on social connection, social isolation, and loneliness among older adults [4,13-19]. Results have been mixed, and the varying study designs, ICT mediums, settings, communication partners (eg, family members, friends, volunteers, or groups), measures, and outcomes make it difficult to draw firm conclusions. One potential explanation for the mixed results on social connection is the lack of attention to social presence within ICT interventions. In a recent systematic review of interactive technology interventions with older adults, few studies had a theoretical basis, and none examined social presence [19].

In information communication technology, social presence is the subjective feeling that other real people are present, involved, and connected within the mediated digital environment, influencing the warmth and personal connection felt during the interaction [20-22]. Satisfaction with ICTs is largely based on the quality of the social presence afforded. With its foundation in theories of interpersonal communication and symbolic interactionism, social presence is strongly correlated with the awareness of others and with a sense of connection with them [23]. Social presence is a transient state that varies with the ICT used, familiarity with the other person, content, environment, and context [21,23,24].

#### What Is Augmented Reality and Why Use It With Older Adults?

Augmented reality (AR) superimposes digital elements onto the real world, allowing real-time interaction between the user and the digital elements. Unlike virtual reality (VR), which immerses the person into a synthetic environment, AR allows the user to maintain visualization of their physical environment [25]. AR terminology and type vary [26-28]; our focus is on head-mounted display AR (HMD-AR), which projects virtual content onto some portion of the real world, also referred to as mixed reality [29,30].

Advantages of HMD-AR over HMD-VR include the following: (1) HMD-AR overlays synthetic elements onto the real world, thus allowing the user to move about safely because room obstacles are apparent; (2) it is less resource intensive because only virtual objects need to be created and overlaid onto a room setting; (3) it provides greater depth perception, enabling the user to see one’s own body, the interacting person’s body, and physical objects, resulting in faster task completion; and (4) it is associated with fewer side effects, including reduced motion sickness, digital eye strain, or isolation effects [30-35]. Importantly, older adults generally accept HMDs [35,36].

With increased computing power in newer AR-based HMD devices, AR is seen as a viable alternative for developing interaction-based interventions for older adults, primarily due to its increased immersion and social presence [22]. The latest AR-based HMDs have increased field of vision, seamless hand and eye tracking, and robust workspace localization. Importantly, users are able to wear these HMDs with their eyeglasses on, which is an important requirement when dealing with older adults.

#### AR Studies Involving Older Adults

Although HMD-AR technology has matured, research examining its applications among adults aged 65 years and older is in its early stages. Existing study designs are primarily nonexperimental, consist of small sample sizes, focus on hardware and software development, and include limited activities, such as park design [37]; exercise, balance training, and fall prevention [38-40]; and cognitive training and e-learning [27,40,41]. One study used augmented VR (a spectrum combining AR and VR elements) to examine a joint meal among 3 older adults [42]. Few studies have involved older adults with cognitive impairment [41].

All these previous studies reveal the promise for HMD-AR; most older adults accepted the technology and experienced few side effects. Suggestions for improvement included a lighter-weight HMD type that fits over eyeglasses, a more realistic avatar design, larger synthetic objects, enhanced user training, and the incorporation of varying levels of difficulty.

### Preliminary Work

As part of the overall grant funding, we conducted several sequential studies before implementing the pilot feasibility and acceptability study.

#### Older and Younger Adults’ Perceptions of AR Photorealistic Avatars as a Viable Medium for Interpersonal Communication

Given that AR as an ICT has been understudied, we examined perceptions of older adults (n=31) and younger adults (n=31) using HMD-AR with photorealistic avatars as a medium for social communication [43]. In addition, we examined the extent to which photorealistic avatars could portray 6 common emotions compared to video clips of a real person.

To assess participants’ perceptions, older adults (n=31) and younger adults (n=31) interacted with a volunteer’s 3D photorealistic avatar using a structured conversational activity through an HMD. Participants rated the quality of HMD-AR communication based on physical and human realism, comfort while talking to the avatar, and the degree of social presence offered by HMD-AR. Participants then identified 6 basic emotions exhibited by (1) video clips of a real person and (2) an animation of their 3D photorealistic avatars via an HMD. Each participant viewed 36 videos and avatar stimuli. Subgroup analyses were conducted by age group.

Participants reported a positive communication experience with the 3D photorealistic avatar, with older adults rating the quality higher than young adults. Ratings were generally lowest for perceptions of how life-like the avatar appeared (68% among younger adults and 61% among older adults). Most participants were able to accurately identify emotions displayed by the 3D photorealistic avatars, although younger adults outperformed older adults (93% accuracy versus 80% accuracy).

#### Cocreation of Collaborative HMD-AR Activities

Given the range of physical and cognitive impairments among older adults, especially for those who reside in LTC settings, we used an iterative participatory design methodology involving LTC older adults (n=8), staff (n=5), and family members (n=2) to develop collaborative HMD-AR activities [44]. Several factors were considered, including older adults’ ability to tolerate HMD-AR for up to 30 minutes without experiencing adverse effects, preferences for HMD-AR activities, and ability to navigate the HMD-AR environment and manipulate AR objects. All iterative participatory design activities took place at an LTC in sequential visits over 6 months. At each visit, participants interacted with the HMD-AR activities and provided feedback that was used to refine the specific modes of interaction, game or activity logic, and user interface elements. We identified the need to tailor AR activities for older adults who experienced a range of physical limitations, including impaired fine motor control (eg, grasping and pinching), cognitive limitations (eg, reduced ability to verbalize thoughts), and sensory impairments (eg, difficulty seeing or reading text). A number of modifications have enabled easier interaction for older adults. Ultimately, 2 collaborative HMD-AR activities were developed for the pilot randomized controlled trial (RCT): fireplace decoration and checkers.

### Specific Aims

The primary aim of this pilot study was to examine the feasibility and acceptability of cocreated collaborative HMD-AR activities using photorealistic avatars. To evaluate the feasibility and acceptability of the study procedures for a future RCT comparing HMD-AR with photorealistic avatars versus 2D ICT (eg, Zoom), we used an RCT design.

## Methods

### Study Design

**Figure 1:**
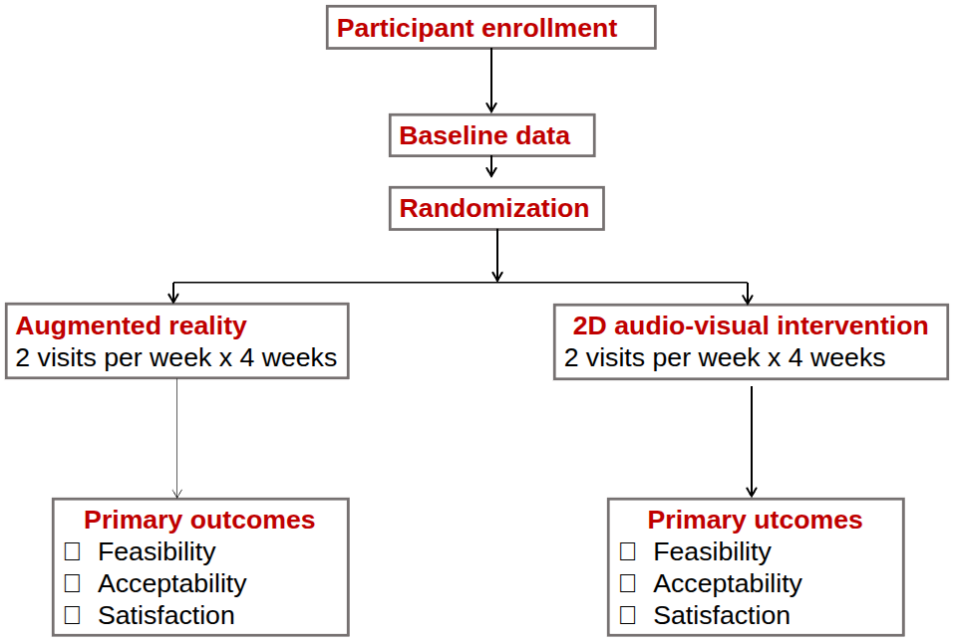
Study flow diagram.

This study was a feasibility and acceptability evaluation of older adults’ use of HMD-AR compared with 2D ICT (ClinicalTrials.gov NCT06179225). To inform a future RCT, we designed the pilot as a nonblinded 2-arm RCT (Figure 1).

Participants were recruited from April 2024 through May 2025. The primary aim was to examine the feasibility and acceptability of the 2 ICTs. We enrolled 8 pairs of older adults and their designated companions (ie, designated family members or friends). After completing baseline assessments, 2 to 4 pairs were enrolled at a time because of logistical constraints. Randomization was conducted at a 1:1 ratio. The intervention consisted of 8 sessions delivered over 4 weeks (2 sessions per week). Baseline evaluations were conducted at week 0. Social presence was evaluated after the second and eighth sessions. Feasibility and acceptability outcomes were evaluated at completion of the study.

### Study Design Rationale

We targeted older adults, with and without cognitive impairment, who resided at home or in LTC settings because of the high prevalence of loneliness or social isolation in these populations, resulting in further cognitive and physical decline [4-7]. Reviews have yielded mixed findings on whether men or women are more likely to report loneliness [4,6,45]. Age does not appear to have an association with levels of social presence in ICT; however, women tend to report higher levels of social presence when using ICT [24].

We used a 2D audio-visual intervention as the comparator because it has been extensively used since the COVID-19 pandemic. Although it provides audio and visual communication, it limits the quality of the interaction and has produced mixed results among older adults residing in the community [4,13-15,46,47]. Our focus is on known family members or close friends because of older adults’ desire to maintain meaningful long-term relationships [48].

We examined feasibility and acceptability because these factors are highly salient for adoption and intervention success [49,50]. An RCT design was chosen for its robustness in conducting future intervention studies. We chose 2 visits per week for a 4-week duration to allow sufficient exposure to assess feasibility and acceptability. There are several HMD products; at the time of this study, we chose HoloLens 2 (Microsoft Corporation) because of its robustness, widespread use (eg, US Army with >80,000 hours of soldier feedback) [51], usability among older adults [52], and ability to fit over eyeglasses.

### Ethical Considerations

This study protocol was approved by the Vanderbilt University Medical Center Institutional Review Board (IRB; 221150). Written informed consent was obtained in person from all participants before data collection and study procedures. To ensure that potential participants understood the study purpose, risks, and benefits, we administered the University of San Diego Brief Protection of Human Subjects Capacity to Consent instrument [53].

Research assistants (RAs) underwent training to recognize signs of frustration, anxiety, or stress displayed by participants; any sign of discomfort resulted in termination of the session. RAs were trained in procedures to protect privacy and confidentiality. Participants were assigned unique study ID numbers, and all deidentified data were stored in a secure password-protected database.

Participants received financial compensation for study activities, including the interactive sessions and data collection procedures, to acknowledge their time and reduce attrition. An external safety officer, designated by the National Institute on Aging (NIA), reviewed and approved the study protocol and informed consent documents before the start of the study. Biannual reports were provided to both the safety officer and the NIA program officer to review progress and any untoward events.

### Conceptual Framework

**Figure 2:**
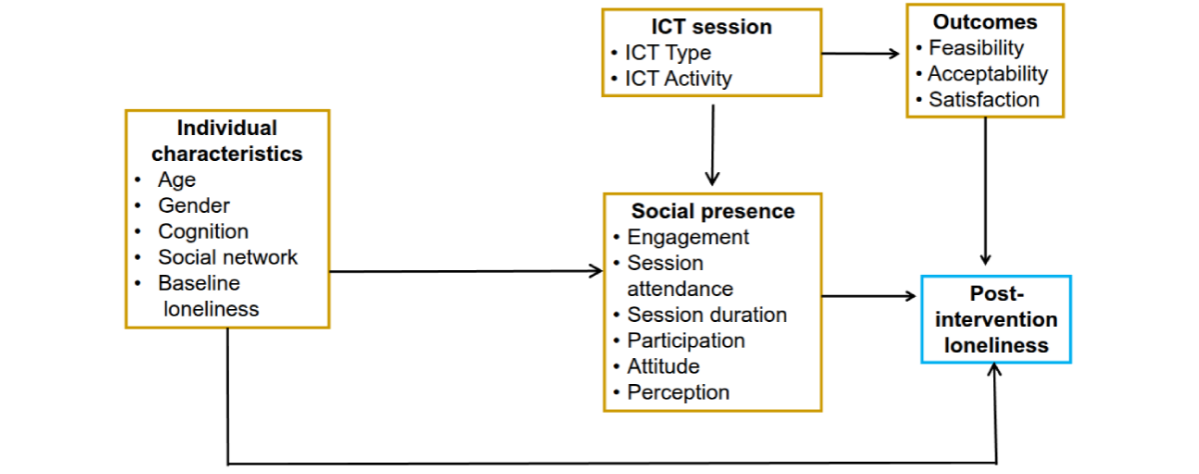
Conceptual framework guiding the study design and intervention. ICT: interactive communication technology.

Several frameworks guided the study (Figure 2). First, social presence theory postulates that the ICT properties and the persons’ perceptions, behaviors, or attitudes regarding the physical presence of other people within the ICT environment affect the quality of the interaction [20,21,23]. We compared 2 ICTs, HMD-AR and 2D audio-video communication. Second, we applied the engagement framework proposed by Cohen-Mansfield [54-56], which emphasizes the role of social interactions, activities, and environment in shaping engagement. Third, the social connection framework by Holt-Lunstad et al [1] guided our focus on the functional and qualitative aspects of ICT visits. Finally, we adapted the unified theory of acceptance and use of technology framework [57] to guide the evaluation of users’ acceptance and use of the ICTs. Individual characteristics such as age, gender, cognition, relationship with designated companion, and social network can impact acceptance and use of ICT [57], the type and quality of engagement [58], and loneliness—our future condition of interest [4].

### Participants and Setting

#### Overview

This study took place in the greater Nashville, Tennessee area. Eligibility criteria for older adults included being aged 60 years or older and living within a 1-hour drive from the principal investigator’s office. If the older adults resided in LTC settings, they must have been residents for 3 months or more. Exclusion criteria included severe cognitive, sensory, or physical impairments that impeded participation; major psychiatric disorders; inability to provide assent; inability to understand or speak English; and acute or terminal illness.

Each older adult recruited 1 companion (family member or close friend). Companions had to be aged 18 years or older and willing to participate in both HMD-AR and 2D audio-video visits and related study procedures. For both older adults and their companions, adequate internet bandwidth was necessary. Eligible older adult–companion pairs were randomized at a 1:1 ratio to ICT type using a computer-generated randomization scheme after baseline measures were completed.

#### Recruitment

Using IRB-approved communication scripts, flyers were posted in public areas, including Vanderbilt University parking garages, local community and older adult centers, churches, and libraries. RAs provided several in-person demonstrations at local centers. Emails were sent to several assisted living facilities located near Vanderbilt University. Interested older adults and family members contacted the investigator team directly. RAs followed the IRB-approved informed consent document to explain the study either in person or by telephone. A standardized screening checklist was completed to ensure eligibility.

#### Sample Size

There is no precise sample size for an early-stage proof-of-concept technology feasibility study. Nielsen and Landauer [59] found that 5 users uncovered 85% of usability problems with the technology. Our target was to enroll 12 pairs of older adults and designated companions for a sample size of 24, as the recommended sample size for pilot studies is 20 to 30 [60,61]. Our final sample of 8 pairs (16 participants) with 8 sessions per pair (64 sessions) was judged sufficient to address the primary aim of feasibility and acceptability of ICT in the home setting.

### HMD-AR and 2D Audio-Visual Interventions

For both interventions, the internet bandwidth within the homes of older adults and family members was measured, and a hot spot was used when necessary to facilitate data streaming. A minimum download speed of 80 Mbps was required for data communication. For each session, a trained RA engineer was physically present in both the older adults’ and companions’ homes to troubleshoot for technological issues during the sessions. The RA trained the participants in establishing the ICT (HMD-AR or 2D audio-visual intervention) connection and assisted participants as needed. The RA remained in the room to monitor for technological issues and maintain field notes. Session duration was at the discretion of the older adult and family member or close friend, but it did not exceed 60 minutes.

### HMD-AR Intervention

#### Creation of Photorealistic Avatars

We created photorealistic avatars for all participants randomized to the HMD-AR study arm. A mobile app, Polycam, was used to take multiple photos of the participant’s face using a 360° view. Polycam constructed a 3D model from these photos that was then used to create a MetaHuman on Unreal Engine, matching the facial geometry and the facial features of the participant. However, MetaHuman Creator is limited in its ability to match the features exactly; therefore, the RAs added manual touch-ups to ensure the avatar looked similar to the participant. After creating the first version of the avatar, a video call was arranged with the participants to share their avatar with them and receive feedback. Participants were able to pick their avatar’s clothing, hair, body shape, and accessories, and apply makeup. If a participant was not satisfied with the likeness of their avatar, the process was repeated, and more manual touch-ups were added. A second meeting was arranged with the participants to share the new version. For most participants, it took 1 meeting to finalize their avatars.

#### Experimental Setup

Participants, in consultation with the RAs, determined the physical space for conducting the sessions. General criteria for physical space were that it was free from clutter, furniture, and objects to allow for clear overlays of virtual objects and photorealistic avatars and located in an area of low traffic to minimize session interruptions. For each participant, equipment included a Kinect camera (Microsoft Corporation), a microphone, an HMD, and a laptop on which the data sharing and the activity ran and were remotely displayed on each participant’s HMD. An Alienware (Dell Technologies Inc) laptop with a 3060 Graphics Processing Unit was used due to the high-performance demand of the AR activity. The RAs ensured that both the older adult and their family members or close friends were connected before moving to an unobtrusive viewing area. Upon completion of the session, the RA dismantled all equipment and cleaned the HMD-AR with disinfectant wipes.

#### Collaborative AR Activity Sessions

Before the start of the experimental sessions, RAs provided one-on-one training and orientation to the HMD-AR to ensure that the participants were able to navigate the system. In addition, a hard-copy, 1-page quick reference guide was provided to each participant. Each older adult–companion pair participated in 2 sessions per week for 4 weeks (total of 8 sessions). Two collaborative AR activities were available to the participants [44]: fireplace decoration and checkers. If the fireplace activity was selected, participants could choose from a variety of virtual objects to decorate the fireplace mantel, hearth, or nearby wall. For the checkers activity, each participant sat at a table, and the checkerboard and checkers appeared as a virtual object in space. Participants were able to see each other’s photorealistic avatars during both activities. Upon completion of each session, the participants would let the RAs know who turned off the AR display and stopped the data communication.

### 2D Audio-Visual Intervention

#### Experimental Setup

Participants, in consultation with the RAs, determined the physical space for conducting the sessions, preferably in an area of low traffic to minimize session interruptions. If the participants were placed in the 2D ICT group, they could use their PC. In the event that participants did not own a device, a laptop was provided for each session. The RAs ensured that both the older adult and their companion were connected before moving to an unobtrusive viewing area. Upon completion of the session, the RAs recovered any loaned devices.

#### Collaborative 2D Audio-Video Sessions

For this study, we used the Zoom videoconferencing application for all sessions. Before the start of the experimental sessions, RAs provided 1:1 training and orientation to using the laptop device, if needed, especially for older adults unaccustomed to tablets or computers and logging in to Zoom. Participants were provided with links to an online checkers game and an online room decoration activity that they could engage in during the session. A 1-page quick reference guide was provided to each participant.

### Ensuring Intervention Fidelity

We incorporated recommendations from the National Institutes of Health Behavior Change Consortium to maximize treatment fidelity across RAs and over time [62]. In brief, we used theoretical models to guide our study, conducted standardized training for RAs with ongoing reviews, and used standardized protocols. Issues were discussed at the weekly investigator-RA meetings. RAs completed field notes after each session, documenting the session date, start and end times, notes on issues encountered during the session, and quotes from participants, and evaluated the amount of effort the older adults exerted to accomplish tasks.

### Data Collection Procedures

Data were collected throughout the experiment from the older adult and their companion, as detailed subsequently. A final interview was conducted at the experiment’s conclusion. All data were collected in person using standardized assessment forms and entered into REDCap (Research Electronic Data Capture; Vanderbilt University) [63], a secure, web-based software platform, and double checked for accuracy. During face-to-face interactions, participants read the questionnaire while the research staff read the questions aloud and documented the responses. Instruments were chosen with established validity and reliability. All data collection forms were scanned and uploaded into REDCap. Table 1 displays the study assessment measures and timing.

**Table 1:** Study assessments and the schedule of evaluations.

Assessment			Enrollment and baseline assessment		Week 1 (sessions 1 and 2)		Week 2 (sessions 3 and 4)		Week 3 (sessions 5 and 6)		Week 4 (sessions 7 and 8)		Week 6 (follow-up: final visit)			
Eligibility review and informed consent			✓													
**Baseline measures**																
	Demographics		✓													
	Physical function: Barthel index^a^		✓													
	**Cognition**															
		AD8 questionnaire		✓												
		Dementia Severity Rating Scale		✓												
		Self-Administered Gerocognitive Examination Questionnaire		✓												
	**Social network**															
		Lubben Social Network Scale for Long-Term Care^b^		✓												
		6-item UCLA^c^ Loneliness Scale		✓												
**Session variables**																
	Attendance				✓		✓		✓		✓					
	Engagement: Observational Measurement of Engagement tool				✓		✓		✓		✓					
	3-item UCLA Loneliness Scale				✓		✓		✓		✓					
	6-item Mutual Awareness Subscale				✓		✓		✓		✓					
**Outcomes**																
	**Feasibility and acceptability**															
		Feasibility of Intervention Measure questionnaire												✓		
		Acceptability of Intervention Measure questionnaire												✓		
	**Exploratory variables**															
		6-item UCLA Loneliness Scale												✓		
		Network Minds Measure of Social Presence												✓		
		Semistructured interviews assessing facilitators and barriers												✓		

^a^Only for participants residing in assisted living facilities.

^b^For participants residing in assisted living facilities, the Lubben Social Network Scale for Long-Term Care (LSNS-LTC) was used for long-term care settings.

^c^UCLA: University of California, Los Angeles.

### Measures

#### Feasibility and Acceptability Outcomes

Feasibility, defined as the practicality of conducting the study [64], was measured by observation and documentation of recruitment and retention rates, data completion, and session attendance (ie, intervention completion). Logistical, technological, and user issues were observed and documented after each session. We measured intervention implementation rates for both study arms as the proportion of successful connections. Feasibility was also measured by the participants’ perceptions of the ease of using the technology using a valid and reliable 4-item questionnaire, the Feasibility Intervention Measure [49]. Each item was rated on a 5-point Likert scale ranging from 1 (completely disagree) to 5 (completely agree). Data were collected during the final 2 weeks after the follow-up visit.

Acceptability, defined as the perception that the intervention (HMD-AR or 2D audio-visual intervention) was agreeable and palatable, was measured using a valid and reliable 4-item questionnaire—the Acceptability Intervention Measure [49]. Each item was rated on a 5-point Likert scale ranging from 1 (completely disagree) to 5 (completely agree). Data were collected 2 weeks after the follow-up visit. Overall satisfaction with the ICT was ascertained at the final session via semistructured interviews.

#### Baseline Variables

For both older adults and companions, baseline data were collected on demographics (age, gender, and race), social network, and loneliness. Social network was assessed using the 12-item Lubben Social Network Scale [65], a self-report measure of social engagement with family and friends. Each item is rated using a 6-point Likert scale ranging from 0 (no monthly contact) to 5 (daily contact); scores range from 0 to 60, with higher scores indicating a greater social network.

For older adults residing in LTC, the Revised Lubben Social Network Scale–Long-Term Care Scale [66] was used; this scale includes an additional 11 items that assess quality and frequency of interactions with other residents and staff members. Loneliness was assessed using the 6-item revised University of California, Los Angeles (UCLA) Loneliness Scale [67], a self-report measure consisting of 6 items rated using a 4-point Likert scale. The score ranges from 6 to 24, with higher scores indicating greater loneliness.

For older adults, cognition was assessed using the Self-Administered Gerocognitive Examination [68,69]. The Self-Administered Gerocognitive Examination is a brief tool that assesses language, reasoning or computation, visuospatial, executive, memory, and orientation domains. Scores range from 0 to 22, with scores greater than 16 suggesting normal cognition. Physical function was assessed via self-report using the Barthel Index [70]. Participants rate their degree of independence in performing 10 activities of daily living (eg, bathing and dressing); scores range from 0 to 100, with higher scores indicating greater independence.

#### ICT Sessions

Immediately after each session, RAs completed a modified Observational Measurement of Engagement [54] instrument to note engagement behaviors, type of activity, level of noise, and lighting. Additional field notes provided contextual information for understanding the older adults’ engagement with their family members across each type of ICT. Participants were asked to complete the 3-item UCLA Loneliness Scale [71].

The Mutual Awareness Subscale (MAS) of the Networked Minds Measure of Social Presence [72] was completed after the second and eighth sessions. The MAS is an 8-item questionnaire rated using a 5-point Likert scale, ranging from 0 (never) to 4 (a great deal); scores range from 0 to 32, with higher scores indicating a greater sense of social presence with one’s partner.

At the last session, participants completed the Networked Minds Measure of Social Presence instrument, consisting of several subscales: MAS (8 items), Perceived Attentional Engagement (6 items), Perceived Emotional Contagion (8 items), Perceived Comprehension (6 items), and Perceived Behavioral Interdependence (6 items). Higher scores indicated greater social presence. Loneliness is the targeted primary outcome for future studies; therefore, participants also completed the 6-item UCLA Loneliness Scale [68]. Finally, the RAs conducted a brief interview using a semistructured questionnaire to elicit participants’ perceptions of facilitators and barriers.

### Analysis Plan

We will produce a CONSORT (Consolidated Standards of Reporting Trials) flowchart to track the progress through the phases of the trial (enrollment, intervention allocation, follow-up, and data analysis). Reasons for noneligibility, nonparticipation, and attrition will be documented and summarized. Feasibility and acceptability (eg, participant recruitment, enrollment, and retention rates) will be descriptively analyzed using frequency distributions and measures of central tendency and dispersion, as appropriate. For participant-completed instruments (eg, social presence, Feasibility Intervention Measure, and Acceptability Intervention Measure), we will examine the extent of missing data to inform the selection of future instruments.

## Results

Funding was received from the NIA in August 2022 and the National Science Foundation in October 2022 (Multimedia Appendices 1 and 2). This study was approved by the local IRB and registered at ClinicalTrials.gov. Following the initial design and testing of the HMD-AR activities, recruitment and data collection for the feasibility and acceptability study began in April 2024 and were completed in May 2025. Of the 8 enrolled older adult and family member pairs, 8 (100%) completed all sessions. Final acquisition of data has been completed, and the data are currently undergoing cleaning. Results are intended to be published in 2026.

## Discussion

This study will assess the feasibility of implementing HMD-AR communication between older adults and family members or close friends and identify key facilitators of and barriers to implementation. This project has several innovations. First, collaborative HMD-AR delivers enhanced social connection for older adults and their family members or friends via a 3D hologram using realistic avatars that are transported to each other’s environments. Most previous work on older adults in AR focuses only on a single user and does not include collaborative aspects. Second, the proposed technology has the capability to map the remote users’ interactions with augmented objects in a chosen environment. Interactions can be mapped efficiently even when the remote and local users’ environments are nonisomorphic. Third, assessing the feasibility and acceptability of collaborative AR among older adults residing alone in the community will be novel to the literature. This work contributes to the knowledge on technology-based interventions targeting loneliness by examining the underlying mechanism of social presence in ICT.

Several limitations exist. It will not be possible to blind participants to the study arms. Attrition can significantly impact studies with small sample sizes. On the basis of our previous experiences, we believe attrition will be low but may occur due to illness or disinterest. We will address attrition through regular communication with the participants and by scheduling sessions at times convenient for participants. Cost and complexity may be issues in applying advanced ICT. We chose to use commercially available, nonproprietary equipment to minimize costs. We plan to make computational algorithms freely available. AR technologies and components are becoming cheaper as demand and applications increase. Almost any new technological intervention plan will introduce additional costs and complexity, both in the home and in the existing system of care; our proposed system is no exception. Whether such an additional burden justifies the new technology is a question that needs to be answered on a case-by-case basis.

Our work will expand on previous studies and focus on HMD-AR to address loneliness. We expect a greater understanding of the feasibility, acceptability, and social presence associated with the use of varying collaborative HMD-AR activities and environments for older adults with different levels of cognitive impairment and their family members or close friends. We also anticipate a greater understanding of the logistics and deployment of this technology in homes and in LTCs.

## Appendix

Multimedia Appendix 1 Peer review report from SPIP - Social Psychology, Personality and Interpersonal Processes Study Section, Risk, Prevention and Health Behavior Integrated Review Group, National Institute on Aging (National Institutes of Health, USA).

Multimedia Appendix 2 Investigator’s response to the peer review report.

## Abbreviations

AR: augmented reality
CONSORT: Consolidated Standards of Reporting Trials
HMD: head-mounted display
HMD-AR: head-mounted display augmented reality
ICT: interactive communication technology
IRB: Institutional Review Board
LTC: long-term care
MAS: Mutual Awareness Subscale
NIA: National Institute on Aging
RA: research assistant
RCT: randomized controlled trial
REDCap: Research Electronic Data Capture
UCLA: University of California, Los Angeles
VR: virtual reality

## Data Availability

Final research data will be uploaded and made available on the website, Open Science Framework [73]. This will occur as our manuscripts are accepted for publication and no later than the online publication date. The engineering know-how for creating the collaborative augmented reality activities and programming techniques will be shared with other researchers through a no-fee research-use only license to be issued upon written request to the principal investigators.
