# Supplementary material for: Feasibility and Acceptability of Collaborative Augmented Reality for Older Adults and Companions: Protocol for a Randomized Controlled Trial
Source: JMIR Res Protoc. 2026 Feb 11;15:e83864. doi: 10.2196/83864 (PMC12936662; doi:10.2196/83864)
Supplement: Multimedia Appendix 1 [file resprot_v15i1e83864_app1.pdf]

SUMMARY STATEMENT
(Privileged Communication)

PROGRAM CONTACT:
Dana Plude
301-435-2309
dana.plude@nih.gov

Release Date: **03/22/2022**
Revised Date:

Application Number: **1 R21 AG078480-01**

Principal Investigators (Listed Alphabetically):

MION, LORRAINE C
SARKAR, NILANJAN (Contact)

Applicant Organization: VANDERBILT UNIVERSITY

Review Group:	**SPIP**<br>**Social Psychology, Personality and Interpersonal Processes Study Section**
Meeting Date:	**02/24/2022**
Council:	**MAY 2022**
Requested Start:	**07/01/2022**
RFA/PA:	**PA20-194**
PCC:	**2BBCHDP**

Project Title:	**Reducing Loneliness of Older Adults in Long Term Care Facilities through Collaborative Augmented Reality**
SRG Action:	**Impact Score:34 Percentile:22 +**
Next Steps:	**Visit https://grants.nih.gov/grants/next_steps.htm**
Human Subjects:	**48-At time of award, restrictions will apply**
Animal Subjects:	**10-No live vertebrate animals involved for competing appl.**
Gender:	**1A-Both genders, scientifically acceptable**
Minority:	**1U-Minorities and non-minorities, scientifically unacceptable**
Age:	**3A-No children included, scientifically acceptable**

Project Year	Direct Costs Requested	Estimated Total Cost
1	150,000	236,771
2	125,000	197,310
TOTAL	275,000	434,081

ADMINISTRATIVE BUDGET NOTE: The budget shown is the requested budget and has not been adjusted to reflect any recommendations made by reviewers. If an award is planned, the costs will be calculated by Institute grants management staff based on the recommendations outlined below in the COMMITTEE BUDGET RECOMMENDATIONS section.

1R21AG078480-01 SARKAR, NILANJAN

INCLUSION OF MINORITIES PLAN UNACCEPTABLE
PROTECTION OF HUMAN SUBJECTS UNACCEPTABLE

RESUME AND SUMMARY OF DISCUSSION: This application proposes to examine the feasibility and acceptability of a head-mounted display augmented reality tool to facilitate older adults' social interaction with their families at long-term care (LTC) facilities. Reviewers agreed that the significance of the proposed work is high because it could provide a solution to the great need of reducing loneliness among older adults at LTC by applying current innovative and cutting-edge technology. The investigative team has complementary expertise in virtual technologies, implementation sciences, nursing, and cognitive functioning, and is experienced working with older adults in LTC settings. Reviewers noted several other strengths, including a strong conceptual framework informed by multiple theories, testing the tool with different levels of cognitive impairment, preliminary data to demonstrate feasibility, and having support from a local LTC facility. Some weaknesses were also noted, including potential confounds in the control group, lacking clarity on the intervention efficacy in addressing loneliness, and other implementation issues. Some reviewers viewed the weaknesses outweigh the strengths, while others remained enthusiastic for its potential impact and clinical implications. Following the discussion, most of the panel concluded that the application would have a medium impact on the field.

DESCRIPTION (provided by applicant): Social connection is a critical health determinant essential in preventing loneliness (subjective feeling of being alone or isolated). Loneliness is common among older adults with adverse consequences: increased risk of mortality, cardiovascular disease, depression, suicide, cognitive and physical decline, reduced quality of life, and increased healthcare utilization. As compared to those residing in the community, long term care (LTC) residents are more likely to experience loneliness (75% versus 43%). The COVID-19 pandemic has exacerbated and intensified the effects of loneliness among LTC older residents. Interactive communication technology (ICT) interventions, such as social media and video-mediated visits, have recently been examined for their effects on loneliness among older adults with mixed results. One potential explanation is the lack of attention to social presence within the various ICTs. Social presence refers to the quality of the ICT property as well as the participant’s perceptions, behaviors or attitudes that other people are physically present in the mediated interaction. Satisfaction with ICTs is based largely on the quality of the social presence afforded. Our long-term goal is to maximize social connection and engagement through the use of head mounted display augmented reality (HMD-AR), thereby reducing loneliness among older adults in LTC. The purpose of this Stage 1 pilot study is to co-create HMD-AR activities with older adults and their families, determine feasibility, acceptability and satisfaction, and explore factors that influence acceptance and usability. The specific aims are 1) to examine the feasibility, acceptability, and satisfaction of co-created collaborative HMD-AR activities versus 2D audio- visual ICT among older adults with and without dementia, their family member and LTC staff; and 2) to explore facilitators and barriers of HMD-AR implementation. We will recruit 8 older adult LTC residents and 8 family members to participate in 5 participatory-research sessions over 6 months to create a menu of collaborative HMD-AR activities. Subsequently, we will enroll 24 older adult LTC residents (8 with normal cognition, 8 with mild cognitive impairment, and 8 with mild dementia) and a designated family member. Each older adult-family member pair will participate in 8 sessions over 4 weeks using a 2-arm RCT design. Participants will be randomized to either 2D audio-visual ICT or HMD-AR. Our central hypothesis is that HMD-AR technology with a high social presence will result in better outcomes than 2D technology with lower social presence. The primary outcome measure is loneliness, measured at baseline, after each session, at Week 4 and at Week 6. Secondary outcomes include feasibility, acceptability and satisfaction collected via qualitative and quantitative measures. Last, one-on-one interviews will be

conducted with older adults, family members and LTC staff to determine barriers and facilitators to HMD-AR use. Findings will inform the design and implementation of a Stage II study using HMD-AR to address loneliness among LTC adults.

PUBLIC HEALTH RELEVANCE: Three out of four older adults who reside in long term care settings experience loneliness that can result in reduced quality of life, depression and further decline. Because families cannot always visit in person, a novel approach to enhance family visits is through the use of head mounted display augmented reality in which the older adult and family member can view photorealistic images of one another. This study will contribute to the development of improved interactive communication technology to link long term care older adults with various family members, with the long-term goal of enhancing quality of life.

CRITIQUE 1

Significance: 3
Investigator(s): 1
Innovation: 1
Approach: 5
Environment: 1

Overall Impact: The purpose of this Stage 1 pilot study is to, first, co-create activities for interactions with head mounted display augmented reality (HMD-AR) in collaboration with stakeholders to be carried out by older adults with and without MCI and dementia, and their families; then examine these activities for feasibility, acceptability, and satisfaction (compared to 2D audiovisual ICT) with an RCT, and finally explore facilitators and barriers of HMD-AR implementation. The applicants posit that these technologies might maximize social connection and engagement, reduce loneliness, among older adults in LTC by increasing participant's perceptions that social partners are physically present with photorealistic images. Overall, this proposal has great strenghts in innovation, team, and environment and has high significance for finding novel ways to combat loneliness in institutions. Before an RCT can be launched, however, applicants need to iron out a few details in the approach and pretest the technology with the target populations. Significance: There is a high need to address loneliness among individuals in LTC and the use of this technology would have high relevance for long-distance caregivers and caregivers unable to visit due to COVID restrictions or other barriers. The conceptual framework is strong and examining the technology with older adults who have different levels of cognitive impairment will support real-world application. However, there is no pretest of the headset with people who have dementia, that would diminish this reviewer's concern about strong negative reactions. Investigators: The team members have complementary skills and expertise of geriatrics, implementation science, quantitative and qualitative methodologies, have worked together on five studies, and appear extremely qualified to do this work. Innovation: Using Collaborative HMD-AR technology with older adults is very unique and testing the underlying mechanism of social presence in interactive communication technology is innovative. Environment: Both Vanderbilt University and the Ohio State University have the lab and grant management resources capacity to run the project. The partnership with the National Health Care Corporation LTC facilities appears strong and able to carry the project. My primary concerns are with the Approach: While the experimental design is typical and the use of low burden data sources and direct observation plus biomarker assessment of engagement are well-considered, there are moderate weaknesses: (1) The 2D control group does not do activities; this could be a confound in the explanation of outcomes, (2) The validity of some of the measures in the Assisted Living/Nursing Home context and with individuals who have mild dementia is not well supported. (3) No attention to how common and uncorrected sensory impairments, and dementia-

related memory loss, decline of motor skills and motor control, and fearful attitudes towards new technologies, especially headsets, will be handled with regard to the experience of the Headset Augmented Reality experience and RCT data collection. (4) Lack of attention to potential negative side effects influence study outcomes. Minor concerns include (1) that assessing cognitive function only at baseline allows for addressing moderation by cognitive group, but not providing a deeper understanding of why the groups differ in their responses (if they do); (2) The selection of staff could affect the resident's comfort level in the testing sessions and thus influence the outcomes. Minority inclusion: Some inconsistencies in the recruitment and inclusion of minorities between text and table need to be cleared up. Human Subjects: It is unclear how potential negative side effects such as panic in a headset, motion sickness and digital eye strain, and disorientation will be addressed in vivo.

1. Significance:

Strengths

- There is a high need to address loneliness among individuals in LTC.
- Need for greater understanding of the logistics and deployment of this technology in LTCs and family homes.
- There is a need to examine older adults with different levels of cognitive impairment and their family members with regard to enhancing communication options when in person meetings are not possible.
- High relevance for long-distance caregivers.
- Strong foundation of previous work using social humanoid robots, virtual reality, and developing multi-modal activities that focus on physical, social, and cognitive aspects of the interaction.
- AR could be a viable method of delivering interaction-based interventions for older adults, due in part to its increased immersion and social presence.
- The study has a strong conceptual framework that integrates theories from social psychology and technology adaptation.

Weaknesses

- Moderate: A pretest with older adults who have dementia and may react negatively to a headset was not mentioned.

2. Investigator(s):

Strengths

- MPI Sarkar directs the Robotics and Automation Laboratory and has a PhD in Mechanical Engineering and Robotics and is experienced in designing socially assistive robots for older adults. He has worked closely with the interdisciplinary team on previous projects.
- The team members have complementary skills and expertise of geriatrics, implementation science, quantitative and qualitative methodologies and have worked together on five studies.
- MPI Mion's background in nursing science, with specific training and expertise in geriatrics and quality improvement and implementation science is also central for this work.
- Co-I Juckett is the outcomes evaluator for a community-engaged project funded through the U.S. Department of Health and Human Service's Administration for Community Living and has experience building academic-clinical and community partnerships and implementation science, which are important for this application.

- Co-I Scharre is Medical Director of the Center for Cognitive and Memory Disorders at The Ohio State University brings clinical expertise in cognitive impairment and experience in long-term care settings.
- Co-I Tan will provide expertise as a methodologist and seasoned statistician in NIH-funded studies.
- Co-I Tate provides nursing expertise in the needs of older patients and their families, symptom recognition and management, communication and cognitive and psychological sequelae of critical illness, and qualitative and mixed methods.

Weaknesses

- None noted.

3. Innovation:

Strengths

- Using Collaborative HMD-AR technology with older adults is very unique.
- Being able to map the remote users' interactions with augmented objects in a chosen environment has not been tried with older adults who have different levels of cognitive impairment.
- Testing the underlying mechanism of social presence in interactive communication technology is novel as well.

Weaknesses

- None noted.

4. Approach:

Strengths

- A computer tablet will be supplied for use during the study for those without computer access.
- Data sources include observations, ICTs and sensor data downloads, interviews and medical record audits conducted by trained research personnel. Instruments were chosen to minimize participant burden.
- Engagement will be assessed via direct observation using a modified Observational Measurement of Engagement (OME) tool and via electrodermal activity (EDA) and heart rate variability (HRV) via the E4 wristband sensor (Empatica Inc., Cambridge MA).

Weaknesses

- Moderate: It is unclear why the 2D control group does not do activities (see Figure 2). This would be a confound in the explanation of outcomes since activities clearly structure an interaction more and can bring greater ease to conversations with older adults who are, for example, angry to be at the home, unable to initiate a conversation, or have difficulty following a conversation.
- Moderate: Validity of some of the measures in the assisted living/nursing home context and with individuals who have mild dementia is not well supported.
- Moderate: Unclear how valid MDS data is in the context of assisted living to quantify physical function, since it is in the interest of the facility to maintain individuals in that context, sometimes for longer than is appropriate.

- Moderate: The applicants need to address how sensory impairments (such as vision and hearing deficit, which are very common in these settings and often uncorrected), memory loss, decline of motor skills and motor control, and fearful attitudes towards new technologies, especially headsets, will be handled with regard to the RCT data collection.
- Moderate: It is unclear how potential negative side effects such as panic in a headset, motion sickness and digital eye strain will be addressed in older adults with dementia, if they occur. Also, I could see older adults with mild dementia becoming confused about where they are after an AR session "in" their care partner's kitchen. This may lead to greater problem behaviors, agitation, or depression in the older adult. What has been done to pre-test for these potential side effects before the RCT and what strategies are in place to prevent or ameliorate these potential outcomes?
- Minor (wording?) The applicants note that "An RCT design with stratified randomization was chosen for its robustness and ability to allow deeper understanding of cognitive function on the outcomes." This framing suggests mediation. However, there is no actual measurement of cognitive function after baseline, only categorization. This may undermine the stated goal of deeper understanding.
- Minor: The protection of staff in the selection and consent processes are well-considered. (Staff must volunteer and directly contact research personnel if they wish to be involved in the study). However, often staff members are assigned to particular residents. If the resident wants to participate, but "their" staff member does not, what are the implications for the resident's comfort level?

5. Environment:

Strengths

- The Vanderbilt Robotics and Autonomous Systems Laboratory has both a lab facility and a clinical assessment and intervention facility located within the Vanderbilt University campus. It includes adequate computing facilities; conference rooms are equipped for teleconferencing and/or videoconferencing technology that allows for inter-site team meetings.
- Both Vanderbilt University and the Ohio State University will provide adequate secretarial and grant management resources for the project.
- National Health Care Corporation LTC facilities, located at Nashville, Tennessee (support letter is attached) will provide physical space to conduct the experiments, a secured storage space for the equipment, time for their support staff, and most importantly, access to the residents to perform the field trials.

Weaknesses

- None noted.

Study Timeline:

Strengths

- The study timeline is adequate, given the experience of the team.

Weaknesses

- None noted.

Protections for Human Subjects:

Unacceptable Risks and/or Inadequate Protections
Data and Safety Monitoring Plan (Applicable for Clinical Trials Only):

Acceptable

- It is unclear how potential negative side effects such as panic in a headset, motion sickness and digital eye strain will be addressed in older adults with dementia, if they occur. Also, I could see older adults with mild dementia becoming confused about where they are after an AR session "in" their care partner's kitchen. This may lead to greater problem behaviors, agitation, or depression in the older adult. What has been done to pre-test for these potential side effects before the RCT and what strategies are in place to prevent or ameliorate these potential outcomes?

Inclusion Plans:

- Sex/Gender: Distribution justified scientifically
- Race/Ethnicity: Distribution not justified scientifically
- For NIH-Defined Phase III trials, Plans for valid design and analysis: Not applicable
- Inclusion/Exclusion Based on Age: Distribution justified scientifically
- The inclusion of minorities says that applicants "will actively recruit all minorities residing in the study site to have adequate representation" but in the tables, only white participants are included in the clinical trial. A rationale is needed for this.

Vertebrate Animals:

Not Applicable (No Vertebrate Animals)

Biohazards:

Not Applicable (No Biohazards)

Resource Sharing Plans:

Acceptable

Budget and Period of Support:

Recommend as Requested

CRITIQUE 2

Significance: 3
Investigator(s): 3
Innovation: 1
Approach: 4
Environment: 1

Overall Impact: This application proposes to test a Stage 1 pilot study to evaluate the feasibility, acceptability, and satisfaction of an augmented reality intervention among older adults in long-term care. This research will administer a two-arm intervention that compares augmented reality to 2D audio-visual communication between an older adult in long-term care and a family member. The ultimate goal is to develop an intervention using augmented reality to reduce loneliness among older adults in long-term care, particularly older adults with cognitive impairments. Strengths of this application include the public health significance of loneliness among residents in long-term care, the focus on three populations in long-term care (older adults with normal cognition, mild cognitive impairment, mild dementia), the strong, integrated theoretical framework guiding the project, and the multiple outcome measures to evaluate feasibility, acceptability, and satisfaction. There are also some limitations to note. First, the sample will be potentially biased toward more high-functioning, socially integrated older adults in long-term care because there will be no selection on loneliness and all participants must have a family member who lives locally in the area. Second, there is little consideration of how the technological barriers may reduce the ultimate utility of the intervention. Third, loneliness is listed as the primary outcome, rather than feasibility, acceptability, and satisfaction [these outcomes are listed as secondary], and the intervention is underpowered to detect either within-person or intervention effects. Fourth, although strong, the research team is large and the expertise in community engagement is not local to where the data collection will take place. These concerns somewhat reduce the potential impact of the proposed work.

1. Significance:

Strengths

- Lack of social connection and loneliness is a significant public health issue in long term care.
- Augmented reality technologies have the potential to increase social interactions between long term care residents and family members and increase social connection.
- Integrates multiple theoretical frameworks into a strong conceptual model to guide the work.

Weaknesses

- The technological barriers to implementation may reduce the ultimate utility of the intervention.
- Engagement may also be a direct predictor of feasibility/acceptability/satisfaction and not just a predictor of (less) loneliness.

2. Investigator(s):

Strengths

- PI Sarkar is a mechanical engineer with extensive experience with developing virtual technologies and the use of assistive technologies with older adults.
- MPI Mion is an expert in nursing and implementation science and has extensive experience working with older adults in the community and long-term care settings.
- Co-I Juckett has expertise in implementation science and experience in evaluating home- and community-based services for older adults.
- Co-I Scharre is Medical Director of the Center for Cognitive and Memory Disorders at The Ohio State University and will provide expertise in cognitive impairment in long-term care settings.
- Co-I Tan is a biostatistician who will provide methodological and statistical guidance for the project.

- Co-I Tate has expertise in qualitative and mixed methods to assess the feasibility and acceptability of interventions.
- Team has a history of successful collaborations.

Weaknesses

- The long-term care facility where participants will be recruited and tested is in Tennessee, but the members of the team with expertise in collaborating with community partners is in Ohio.
- The team is large with overlapping expertise.

3. Innovation:

Strengths

- The use of augmented reality among residents of long-term care to reduce loneliness.
- The development of a detailed theoretical model that posits the mechanisms of action of the intervention.

Weaknesses

- None noted.

4. Approach:

Strengths

- The inclusion of three populations in long term care: older adults with intact cognition, older adults with mild cognitive impairment, and older adult with mild dementia.
- The manipulation (augmented reality versus 2D audio-visual) and measurement of social presence.
- Support from a local long-term care facility ensures access to the population of interest and support for the research.

Weaknesses

- The sample will most likely be high functioning with relatively low levels of loneliness. Loneliness in the sample is likely to be relatively low unless participants are specifically sampled for higher loneliness.
- Requires multiple cameras in the family member's home and the permission of the family member to allow researchers inside their home to place the cameras in the designated room.
- Since researchers need to enter the family members' home, all older adult participants must have family living nearby. This population is less likely to be socially isolated and lonely than older adults in long term care who do not have family living in the same city.
- Does not include education as an individual characteristic (Figure 1). Education is likely to contribute to how participants engage with and accept the technology.
- The analytic plan is vague, particularly comparisons between the two conditions for the quantitative measures.
- The proposed facilitators and barriers for Aim 2 are vague. In addition to analyzing open-ended responses (which is important), there are other factors that could be considered a priori as

potential facilitators and barriers to measure and test (e.g., education, comfort with technology, family member living situation, etc.).

- Loneliness is listed as the primary outcome, with feasibility, acceptability, and satisfaction as secondary outcomes (4.2. Outcome Measures). Yet, the aims are focused entirely on feasibility, acceptability, and satisfaction and facilitators and barriers and not loneliness. The sample is not powered to detect either changes in loneliness within condition or an interaction with condition on change in loneliness. Although the ultimate goal of this work is an intervention for loneliness, the current design is not sufficient to evaluate change and thus should focus more on the seemingly stated goal of determining feasibility, acceptability, and satisfaction of augmented reality.

5. Environment:

Strengths

- The environment for this work is excellent.

Weaknesses

- None noted.

Study Timeline:

Strengths

- The timeline is reasonable.

Weaknesses

- None noted.

Protections for Human Subjects:

Acceptable Risks and/or Adequate Protections

- The protection of human subjects is adequate.

Data and Safety Monitoring Plan (Applicable for Clinical Trials Only):

Acceptable

- The DSMP is reasonable.

Inclusion Plans:

- Sex/Gender: Distribution justified scientifically
- Race/Ethnicity: Distribution not justified scientifically
- For NIH-Defined Phase III trials, Plans for valid design and analysis: Not applicable
- Inclusion/Exclusion Based on Age: Distribution justified scientifically
- 100% of participants in the trial will be white. It seems like more effort could be made to recruit at least some participants of color.

Vertebrate Animals:

Not Applicable (No Vertebrate Animals)

Biohazards:

Not Applicable (No Biohazards)

Resource Sharing Plans:

Not Applicable (No Relevant Resources)

Budget and Period of Support:

Recommend as Requested

CRITIQUE 3

Significance: 1
Investigator(s): 1
Innovation: 1
Approach: 1
Environment: 1

Overall Impact: This is an extremely innovative and cutting-edge, yet carefully designed, study with strong clinical implication for addressing loneliness in older adults with and without dementia. The investigator team is led by strong technical and clinical MPIs with a long-standing collaboration record. The conceptual framework is comprehensive for understanding multiple dimensions and systems affecting loneliness; the study design for exploring multiple variables with qualitative interviews focusing on feasibility and acceptability is appropriate. The preliminary data, even though not required for R21, is very strong to support the feasibility of its use in aging populations. The study, if successful, will have substantial impact on the field.

Study Timeline:

Strengths

- Reasonable.

Weaknesses

- None noted by reviewer.

Protections for Human Subjects:

Acceptable Risks and/or Adequate Protections

Data and Safety Monitoring Plan (Applicable for Clinical Trials Only):

Not Applicable (No Clinical Trials)

Inclusion Plans:

- Sex/Gender: Distribution justified scientifically
- Race/Ethnicity: Distribution justified scientifically

- For NIH-Defined Phase III trials, Plans for valid design and analysis: Not applicable
- Inclusion/Exclusion Based on Age: Distribution justified scientifically

Vertebrate Animals:
Not Applicable (No Vertebrate Animals)

Biohazards:
Not Applicable (No Biohazards)

Resource Sharing Plans:
Not Applicable (No Relevant Resources)

Budget and Period of Support:
Recommend as Requested

THE FOLLOWING SECTIONS WERE PREPARED BY THE SCIENTIFIC REVIEW OFFICER TO SUMMARIZE THE OUTCOME OF DISCUSSIONS OF THE REVIEW COMMITTEE, OR REVIEWERS' WRITTEN CRITIQUES, ON THE FOLLOWING ISSUES:

PROTECTION OF HUMAN SUBJECTS: UNACCEPTABLE
The committee noted that human subject protections regarding potential negative side effects of using the AR headset was inadequately described.

INCLUSION OF WOMEN PLAN: ACCEPTABLE

INCLUSION OF MINORITIES PLAN: UNACCEPTABLE
The committee noted that the planned enrollment of nearly all White participants was insufficiently justified.

INCLUSION ACROSS THE LIFESPAN: ACCEPTABLE

COMMITTEE BUDGET RECOMMENDATIONS: The budget was recommended as requested.

Footnotes for 1 R21 AG078480-01; PI Name: SARKAR, NILANJAN

+ Derived from the range of percentile values calculated for the study section that reviewed this application.

NIH has modified its policy regarding the receipt of resubmissions (amended applications).See Guide Notice NOT-OD-18-197 at https://grants.nih.gov/grants/guide/notice-files/NOT-OD-18-197.html. The impact/priority score is calculated after discussion of an application by averaging the overall scores (1-9) given by all voting reviewers on the committee and multiplying by 10. The criterion scores are submitted prior to the meeting by the individual reviewers assigned to an application, and are not discussed specifically at the review meeting

or calculated into the overall impact score. Some applications also receive a percentile ranking. For details on the review process, see http://grants.nih.gov/grants/peer_review_process.htm#scoring.

MEETING ROSTER

Social Psychology, Personality and Interpersonal Processes Study Section
Risk, Prevention and Health Behavior Integrated Review Group
CENTER FOR SCIENTIFIC REVIEW
SPIP
02/24/2022 - 02/25/2022

Notice of NIH Policy to All Applicants: Meeting rosters are provided for information purposes only. Applicant investigators and institutional officials must not communicate directly with study section members about an application before or after the review. Failure to observe this policy will create a serious breach of integrity in the peer review process, and may lead to actions outlined in NOT-OD-14-073 at https://grants.nih.gov/grants/guide/notice-files/NOT-OD-14-073.html, NOT-OD-15-106 at https://grants.nih.gov/grants/guide/notice-files/NOT-OD-15-106.html, and NOT-OD-18-115 at https://grants.nih.gov/grants/guide/notice-files/NOT-OD-18-115.html, including removal of the application from immediate review.

CHAIRPERSON(S)

KRUEGER, ROBERT F, PHD
DISTINGUISHED MCKNIGHT UNIVERSITY PROFESSOR
DEPARTMENT OF PSYCHOLOGY
UNIVERSITY OF MINNESOTA
MINNEAPOLIS, MN 55455

MEMBERS

BEACH, LAUREN BRITTANY, PHD *
RESEARCH ASSISTANT PROFESSOR
DEPARTMENT OF MEDICAL SOCIAL SCIENCES
ASSOCIATE DIRECTOR OF THE EVALUATION, DATA
INTEGRATION, AND TECHNICAL ASSISTANCE
NORTHWESTERN UNIVERSITY
CHICAGO, IL 60611

BERG, CYNTHIA A, PHD
DISTINGUSHED PROFESSOR AND DEAN
DEPARTMENT OF PSYCHOLOGY
UNIVERSITY OF UTAH
SALT LAKE CITY, UT 84112

BIRDITT, KIRA S, PHD
RESEARCH ASSOCIATE PROFESSOR
THE INSTITUTE FOR SOCIAL RESEARCH
UNIVERSITY OF MICHIGAN
ANN ARBOR, MI 48106

BOUCHARD, ELIZABETH, PHD
PROFESSOR
DEPARTMENT OF CANCER PREVENTION AND CONTROL
SENIOR VICE PRESIDENT
COMMUNITY OUTREACH AND ENGAGEMENT
ROSWELL PARK COMPREHENSIVE CANCER CENTER
BUFFALO, NY 14263

BOWLING, JESSAMYN *
ASSISTANT PROFESSOR
DEPARTMENT OF PUBLIC HEALTH SCIENCES
COLLEGE OF HEALTH AND HUMAN SERVICES
UNIVERSITY OF NORTH CAROLINA AT CHARLOTTE
CHARLOTTE, NC 28223

COIFMAN, KARIN GALIAH, PHD
ASSOCIATE PROFESSOR
DEPARTMENT OF PSYCHOLOGY
KENT STATE UNIVERSITY
KENT, OH 44242

FAGUNDES, CHRISTOPHER PAUL, PHD
ASSOCIATE PROFESSOR
DEPARTMENT OF PSYCHOLOGICAL SCIENCES
RICE UNIVERSITY
HOUSTON, TX 77005

FREDRIKSEN-GOLDSEN, KAREN ILENE, PHD *
PROFESSOR
SCHOOL OF SOCIAL WORK
UNIVERSITY OF WASHINGTON
SEATTLE, WA 98195

GREENE, KATHRYN L, PHD *
PROFESSOR
SCHOOL OF COMMUNICATION
AND INFORMATION
RUTGERS, THE STATE UNIVERSITY OF NEW JERSEY
NEW BRUNSWICK , NJ 08901

HALEY, WILLIAM E, PHD *
PROFESSOR
SCHOOL OF AGING STUDIES
UNIVERSITY OF SOUTH FLORIDA
TAMPA, FL 33620

HINES, DENISE ANN, PHD *
ASSOCIATE PROFESSOR
DEPARTMENT OF SOCIAL WORK
COLLEGE OF HEALTH AND HUMAN SERVICES
GEORGE MASON UNIVERSITY
FAIRFAX, VA 22030

INFURNA, FRANK JOHN, PHD
ASSOCIATE PROFESSOR
DEPARTMENT OF PSYCHOLOGY
ARIZONA STATE UNIVERSITY
TEMPE, AZ 85287

JOSEPH, NATARIA TENNILLE, PHD *
ASSOCIATE PROFESSOR
DEPARTMENT OF PSYCHOLOGY
SOCIAL SCIENCE DIVISION
PEPPERDINE UNIVERSITY
MALIBU, CA 90263

KIM, YOUNGMEE, PHD
PROFESSOR
DEPARTMENT OF PSYCHOLOGY
UNIVERSITY OF MIAMI
CORAL GABLES, FL 33146

KRENDL, ANNE CATHERINE, PHD *
ASSOCIATE PROFESSOR
DEPARTMENT OF PSYCHOLOGICAL AND BRAIN SCIENCES
INDIANA UNIVERSITY BLOOMINGTON
BLOOMINGTON, IN 47405

LIN, FENG VANKEE, PHD
CLINICAL PROFESSOR
DEPARTMENT OF PSYCHIATRY AND BEHAVIORAL
SCIENCES
STANFORD UNIVERSITY
STANFORD, CA 94305

LITT, DANA M, PHD *
ASSOCIATE PROFESSOR
DEPARTMENT OF HEALTH BEHAVIOR AND HEALTH
SYSTEMS
SCHOOL OF PUBLIC HEALTH
UNIVERSITY OF NORTH TEXAS HEALTH SCIENCE CENTER
FORT WORTH, TX 76107

LITZELMAN, KRISTIN R, PHD *
ASSOCIATE PROFESSOR
DEPARTMENT OF HUMAN DEVELOPMENT AND
FAMILY STUDIES
SCHOOL OF HUMAN ECOLOGY
UNIVERSITY OF WISCONSIN, MADISON
MADISON, WI 53706

MAZOR, KATHLEEN MICHELE, EDD *
PROFESSOR OF MEDICINE
ASSOCIATE DIRECTOR
MEYERS PRIMARY CARE INSTITUTE
UNIVERSITY OF MASSACHUSETTS CHAN MEDICAL SCHOOL
WORCESTER, MA 01605

MUSCI, RASHELLE JEAN, PHD *
ASSOCIATE PROFESSOR
DEPARTMENT OF MENTAL HEALTH
BLOOMBERG SCHOOL OF PUBLIC HEALTH
JOHNS HOPKINS UNIVERSITY
BALTIMORE, MD 21205

PACHANKIS, JOHN EDWARD, PHD
PROFESSOR
DEPARTMENT OF SOCIAL AND BEHAVIORAL SCIENCES
YALE SCHOOL OF PUBLIC HEALTH
YALE UNIVERSITY
NEW HAVEN, CT 06520

POTEAT, TONIA C, PHD
ASSOCIATE PROFESSOR
DEPARTMENT OF SOCIAL MEDICINE
SCHOOL OF MEDICINE
UNIVERSITY OF NORTH CAROLINA AT CHAPEL HILL
CHAPEL HILL, NC 27599

REED, REBECCA G, PHD *
ASSISTANT PROFESSOR
DEPARTMENT OF PSYCHOLOGY
UNIVERSITY OF PITTSBURGH
PITTSBURGH, PA 15260

SALAS-WRIGHT, CHRISTOPHER PATRICK, PHD
PROFESSOR AND ASSISTANT DEAN, DOCTORAL PROGRAM
SCHOOL OF SOCIAL WORK
BOSTON COLLEGE
CHESTNUT HILL, MA 02467

SAXBE, DARBY E, PHD *
ASSOCIATE PROFESSOR
DEPARTMENT OF PSYCHOLOGY
UNIVERSITY OF SOUTHERN CALIFORNIA
LOS ANGELES, CA 90089

SCOTT, STACEY BETH, PHD
ASSOCIATE PROFESSOR
DEPARTMENT OF PSYCHOLOGY
STONY BROOK UNIVERSITY
STONY BROOK, NY 11794

SIEGEL, JASON T, PHD *
PROFESSOR
DEPARTMENT OF PSYCHOLOGY
SCHOOL OF SOCIAL SCIENCE, POLICY AND EVALUATION
CLAREMONT GRADUATE UNIVERSITY
CLAREMONT, CA 917111

SUTIN, ANGELINA R, PHD
PROFESSOR
DEPARTMENT OF BEHAVIORAL SCIENCES AND
SOCIAL MEDICINE
COLLEGE OF MEDICINE
FLORIDA STATE UNIVERSITY
TALLAHASSEE, FL 32306

SÖRENSEN, SILVIA, PHD *
ASSOCIATE PROFESSOR
DEPARTMENT OF COUNSELING & HUMAN DEVELOPMENT
WARNER SCHOOL FOR EDUCATION AND HUMAN
DEVELOPMENT
UNIVERSITY OF ROCHESTER
ROCHESTER, NY 14647

MAIL REVIEWER(S)

DALKE, KATHARINE B
ASSISTANT PROFESSOR
DEPARTMENT OF PSYCHIATRY AND
BEHAVIORAL HEALTH
PENNSYLVANIA STATE UNIVERISTY
UNIVERSITY PARK, PA 16802

SCIENTIFIC REVIEW OFFICER

LUN, JANETTA, PHD
SCIENTIFIC REVIEW OFFICER
CENTER FOR SCIENTIFIC REVIEW
NATIONAL INSTITUTE OF HEALTH
BETHESDA, MD 20892

EXTRAMURAL SUPPORT ASSISTANT

ADEBONA, ADEBIMIBOLA O
LEAD EXTRAMURAL SUPPORT ASSISTANT
CENTER FOR SCIENTIFIC REVIEW
NATIONAL INSTITUTES OF HEALTH
BETHESDA, MD 20892

* Temporary Member. For grant applications, temporary members may participate in the entire meeting or may review only selected applications as needed.

Consultants are required to absent themselves from the room during the review of any application if their presence would constitute or appear to constitute a conflict of interest.
