# Supplementary material for: Feasibility and Acceptability of Collaborative Augmented Reality for Older Adults and Companions: Protocol for a Randomized Controlled Trial
Source: JMIR Res Protoc. 2026 Feb 11;15:e83864. doi: 10.2196/83864 (PMC12936662; doi:10.2196/83864)
Supplement: Multimedia Appendix 2 [file resprot_v15i1e83864_app2.docx]

**RESPONSE TO SUMMARY STATEMENT**

**PI**: Sarkar, Nilanjan (contact); Mion, Lorraine C.

**Grant No.** 1 R21 AG078480-01

**Grant Title**: Reducing loneliness of older adults in long term care facilities through collaborative augmented reality

**Overview of Project**

This interdisciplinary proposal focuses on the design and evaluation of collaborative head mounted display augmented reality (HMD-AR) as an interactive communication technology for older adults residing in long term care communities especially for those with AD and their family members. The long term goal is to enhance social connection and engagement through HMD-AR, thereby mitigating loneliness. The specific aims of this Stage I pilot study are: Aim 1: To examine the feasibility, acceptability, and satisfaction of co-created collaborative HMD-AR activities versus 2D communication (e.g., Zoom) among older adults with and without AD, their family member and long term care staff. In the first phase of Aim 1, a participatory design approach with 8 older adults, their family and 5 LTC staff will establish a menu of HMD-AR activities. In the second phase of Aim 1, we will enroll 24 older adults, stratified by cognitive impairment, and their family member to take part in a 4 week RCT with two arms: HMD-AR or 2D audiovisual communication. Aim 2: To explore facilitators and barriers of HMD-AR implementation using qualitative methods. This study will contribute to the development of advanced intelligent technology as an effective approach to engage older adults with the long-term goal of enhancing function and quality of life.

**Response to the Resume and Summary**

The **Resume and Summary Discussion** enumerated the strengths of our proposal including the significance, innovative and cutting-edge technology, complementary expertise of the interdisciplinary team and experience working with older adults in LTC settings, strong conceptual framework, testing the tool with different levels of cognitive impairment, preliminary data to demonstrate feasibility, and support from a local LTC facility. Some weaknesses were noted including *potential confounds in the control group, lacking clarity on intervention efficacy in addressing loneliness, and implementation issues*.

***Potential confounds in the control group.***  A potential confounder is the joint older adult–family member activity during the interactive communication technology visit (HMD-AR versus 2D audio-visual). Although it was not clearly mentioned in the proposal, activities as desired by the control group that take place will be part of the session data collection. In addition, we will include the joint meal activity as described for the HMD-AR group. A second potential confounder for the overall study is the extent to which sensory impairments, memory loss, decline in motor skills and control, education, digital proficiency (defined as prior experience with tablet or smartphone), and fearful attitudes towards new technologies may influence the outcomes. First, we will stratify enrollment by level of cognition, a major confounding variable, to ensure equal distribution between the two groups. For the remaining variables, we will collect the information at baseline prior to randomization. After randomization, we will add an exclusion criterion to assess older adults’ comfort and ability to use the technology (HMDs for intervention group; headset with monitor screen for 2D audio). Those who are unable to tolerate the technology will be excluded; this information will be kept as part of feasibility measures. We will evaluate the success of randomization by comparing the characteristics of the two groups and adjusting for confounding as appropriate in the data analysis.

***Lacking clarity on intervention efficacy in addressing loneliness.*** We agree with the reviewers that the pilot nature of the work does not allow for assessment of intervention efficacy in addressing loneliness. Thus we are changing the primary outcome to that of feasibility, acceptability and satisfaction (previously identified as secondary outcomes). We are not indicating a level of loneliness as an inclusion criterion. However, the study measures will allow us to estimate loneliness longitudinally at each time point and produce trend plots by intervention groups. Mixed-effects linear regression will be used to model loneliness as a linear function of group (AR intervention vs. 2D audio-visual control), time (baseline vs. week 4 vs. week 6) and group by time interaction, adjusting for within-subject clustering from repeated measures and potential covariates. From the model, we will derive contrast estimates of both within- and between-group differences in the change of loneliness from baseline. These findings will inform a larger RCT with loneliness as the targeted primary outcome.

***Implementation issues.*** Several concerns were raised regarding implementation. One was the concern of technological barriers to implementation. Two technological barriers may be present. First, internet/broadband access may be lacking in some areas; however a 2021 PEW Foundation study reported that 77% of households in the U.S. have high-speed broadband service^1^ and the U.S. Census Bureau reported that 92% of households had at least one type of computer and 85% had a broadband internet subscription.^2^ We anticipate that access will continue to grow throughout the U.S. Cost is the second potential barrier, but this too has been decreasing for HMDs and Kinect cameras, especially with the proliferation of VR video gaming. As we are experiencing an AI revolution across society, these technological barriers are bound to be less of an impediment due to expansion of internet access and reduction of technology development cost. This R21 grant will allow us to find further solutions to reduce technological barriers.

A second potential implementation issue is with the families. Families must provide permission for cameras and such permission will be part of our inclusion criteria. At a stakeholder meeting, families expressed interest and acceptance of cameras, many of whom already use security cameras. The global smart home security camera market size was valued at USD 3.71 billion in 2019 and is expected to grow at a compound annual growth rate (CAGR) of 15.7% from 2020 to 2027.^3^ For this pilot, we focus on those within driving distance of Vanderbilt University for logistic purposes, i.e., setting up and testing the equipment. Findings will help us create manuals for families to install the equipment on their own in future studies, which will remove any distance barrier for technology implementation.

A third issue raised is that team members with expertise in collaborating with community partners are in Columbus, Ohio, while the pilot study will take place in the Nashville area. PI Sarkar at Vanderbilt has been successfully collaborating with LTC community partners for >5 years. In addition, in order to assuage any concerns, we plan to include a new co-I, Dr. Cathy Maxwell, a nursing faculty member at Vanderbilt University, to the Nashville team; Dr. Maxwell has years of experience and contact with the local LTC communities. The OH team members have been collaborating with PI Sarkar and Nashville area LTCs for several years. OH investigators have frequent and routine video meetings as well as several members (Mion and Tate) make in-person visits. Note that the drive between Columbus and Nashville is only 6 hours, allowing for flexibility in making arrangements for visits based on times most suitable for the community partners.

**Scientific Review Officer Summary Issues.**

**1. Protection of Human Subjects: Unacceptable.** The committee noted that human subject protections regarding potential negative side effects of using the AR headset was inadequately described.

Response**:** Since the submission of this proposal we have assessed the use and acceptability of HMD-AR with 9 older adults, 6 in assisted living and 3 in independent living settings. Ages ranged from 67 – 92 (mean 79.0); two were men. None had ever used HMDs. Each person wore the HMD for 20 minutes and participated in 3 AR activities: painting, playing a keyboard, and interacting with a hummingbird. Participants rated the experience using a 5-point Likert response: enjoyed the AR activities (4.3), comfort level wearing the HMD (4.4), nausea or dizziness (none-5), and would participate again (4.7).

To address this concern, we will use this 20-minute pretest of the equipment prior to implementation of the intervention and will exclude those who have untoward effects. For those who enroll in the study, a trained research staff and an LTC staff member will be present during each session to monitor the older adult’s responses and to end the session upon any behavioral displays or vocalization of discomfort. Upon completion of each session, the staff member and research staff will de-brief the older adult and re-orient them to their environment.

**2. Inclusion of minority plans.** The committee noted that the planned enrollment of nearly all White participants was insufficiently justified.

Response: We concur with this observation. The NHC Place on the Trace has a predominantly White resident population. Since the submission of the proposal we have made further contact with local LTC communities that have a more diverse and minority resident population, Schrader Acres Assisted Living (34 beds; 100% African American), and Knowles Home Assisted Living (100 beds; 40% African American). This will allow us access to recruit participants of color. Our goal is to mirror the Nashville area census reflecting 66% White and 34% minorities. We will oversample minority residents to achieve this distribution and ensure that our technology is feasible, acceptable, and satisfactory to racial/ethnic groups at great risk for health disparities.

**References**:

1. Pew Research Center. Internet/Broadband Fact Sheet. April 7, 2021. <https://www.pewresearch.org/internet/fact-sheet/internet-broadband/>

2. United States Census Bureau. Computer and Internet Use in the United States: 2018. April 21, 2021. <https://www.census.gov/newsroom/press-releases/2021/computer-internet-use.html>

3. <https://www.grandviewresearch.com/industry-analysis/smart-home-security-camera-market>. [**GVR Report cover**](https://www.grandviewresearch.com/industry-analysis/smart-home-security-camera-market)

Smart Home Security Cameras Market Size, Share & Trends Analysis Report By Product (Wired, Wireless), By Application (Doorbell Camera, Indoor Camera, Outdoor Camera), By Region, And Segment Forecasts, 2020 - 2027
